# Baby Brains

**Published:** 1890-08-02

**Authors:** 


### The Doctor's Pulpit.

### BABY BRAINS.

In the year 1834 the *Beagle* visited Port Famine, and Mr. Darwin reports thus of the natives: "During our stay the Fuegians twice came out and plagued us. As there were many instruments, clothes, and men on shore, it was thought necessary to frighten them away. The first time a few great guns were fired when they were far distant. It was most ludicrous to watch through a glass the Indians, as often as the shot struck the water, take up stones, and as a bold defiance throw them towards the ship, though about a mile and a half distant. A boat was then sent with orders to fire a few musket shots wide of them. The Fuegians hid themselves behind the trees, and for every discharge of the muskets they fired their arrows; all, however, fell short of the boat, and the officer, as he pointed at them, laughed. This made the Fuegians frantic with passion, and they shook their mantles in vain rage. At last, seeing the balls cut and strike the trees they ran away, and we were left in peace and quietness. During the former voyage the Fuegians were here very troublesome, and to frighten them a rocket was fired at night over their wigwams; it answered effectually, and one of the officers told me that the clamour first raised, and the barking of the dogs, was quite ludicrous in contrast with the profound silence which in a minute or two afterwards prevailed. The next morning not a single Fuegian was in the neighbourhood."

This brief history paints a picture of grown-up men, a certain proportion of whom would be middle-aged or old, behaving, in circumstances of novelty and apparent danger, exactly as uneducated children or vicious and spiteful monkeys might have behaved. Now, conduct which is perfectly spontaneous manifests the actual condition of the brain and character much more decidedly than conduct which is carefully calculated beforehand. The stage Richard III. or Shylock may be an exceedingly mild and inoffensive person in private life. Some not inconsiderable people, for example, the late Laurence Oliphant, Sir Edwin Arnold, Mr. Robert Louis Stevenson, and others, appear to be too much enamoured of man "in a state of nature," and seem to show both by precept and example that Western civilisation is by no means what they regard as an unmixed good. Many other persons, whose names are not yet publio property, have also a decided hankering after this same "state of nature." In our judgment this same "state of nature" labours under the disadvantage of being mostly inconsequent and often brutal. We who live in the midst of civilisation sometimes have mental and moral "corns," and occasionally get them trod upon. That makes us howl a good deal, and establishes in us a kind of half conviction that the disadvantages of civilisation much outweigh the advantages; and an exceedingly stupid, childish, and intellectually contemptible impression it is.

There is very solid ground for believing that sound education combined with worthy morals promotes the highest development of man, and leads to the greatest happiness. From a medical standpoint, therefore, sound education and high morals take rank among the first of physiological advantages. The philosophic doctor is bound to regard everything which opposes them as an enemy to be fought against and, if possible, driven out of the world. If, for example, the larger half of our countrymen and countrywomen could be suddenly reduced to the "baby-brained" level of Darwin's Fuegians, it would be one of the greatest calamities that could possibly be conceived; and every patriotic lover of his country would feel bound to devote the whole energy of his mind to the intellectual improvement of his fellows. We are quite safe from any danger of that sort, thinks the average reader. But is that really so? Are there not among us any grown-up persons whose brain is in the embryonic and "baby" condition of the brains of the Fuegians? The sober truth is that there are large numbers of persons of both sexes, of all ranks, and of every degree of education, who, for practical purposes, are as morally inconsequent and as intellectually ridiculous as the Fuegians who excited Darwin's notice and contempt.

Take, for example, the inhospitality of some persons to new ideas. It is impossible to believe of many a man that he has a living soul. The impression he gives is that his mind is a kind of machine like the mechanical pillars at the railway stations, made to admit a penny and to work in obedience to its impact, but to reject with disdain or with solemn immobility every other kind of coin whatsoever. So the mental mechanism of many people will receive the "pennies" that they have been trained to receive, or that their grandfathers or great-grandfathers received before them; but if they are offered anything with which they are unfamiliar they cast it away with contempt, or hear it with the silence of unconquerable imbecility. The brain of such people is absolutely embryonic. Some of them have passed from the governess to the preparatory school, and from the preparatory to the public school, and thence to the university. But none of these occasions of mental stimulation have resulted in any maturing or developing of the intellect. As they were in the baby stage, so they are now, and ever will be, world without end. "Such men are dangerous;" much more dangerous than Cassius, with his "lean and hungry look." From a physiological and scientific standpoint the greatest of all dangers to the human creature are intellectual stupidity and deficient moral energy. He who casts away from him all new ideas as the mechanical distributor of sweets casts away everything but pennies is in the last and most hopeless stage of mental and moral decrepitude.

The wilfulness of a certain class of women, a wilfulness which some men fools are silly enough to admire, furnishes us with a very familiar example of embryonic and "baby" brains. It may be pretty in the child to pout and insist upon its own way in every trifle, but in the grown-up woman it is simply idiotic. The Puritans no doubt mixed with their wholesome discipline a good deal of superfluous and unlovely peculiarity of manner. But the demurest Puritanism that the soberest girl in England could be persuaded to adopt was infinitely to be preferred to the shallow affectations and ill-bred self-assertions of the pouting and artificial Miss of the present. There is good reason to hope that in no long time undisciplined wilfulness and uninstructed ignorance will be considered as contemptible and as shameful in women as in men. It would not be fair to apportion all or even the greater part of the blame of the mental deficiencies of the less robust sex to women themselves. Men seem to have always inclined to make either toys or slaves of the sex. But now that the more courageous and strong among women are claiming both to be thinkers and to originate action, the rest of their sisters will deserve to be for ever regarded as hopelessly inferior to man if they do not stand shoulder to shoulder in the conflict for place and recognition which the worthiest of their order are waging. Woman is in many circumstances both lovely and delightful, but never so lovely and delightful as when she adds to her beautiful face and form a sweet disposition and a cultured mind. If ever she is superior to man it is then.

Examples of what is meant by "baby brains" might be multiplied indefinitely from among all sorts and conditions of men. The mental state indicated by the opprobrious words is thoroughly discreditable, and as injurious as it is unworthy. It is the manifest design of nature that when the body is of full size the brain shall be of full capacity, and that there shall always be on a man's shoulders a man's head. Much study may be a weariness to the flesh, but much stupidity is certainly a disgrace to the spirit. Nature has laid upon us all the burden of intellectuality and of divine morality. Physiology shows that he who refuses to bear that burden with a high and manly pride and courage has his proper place among those creatures of undeveloped brain who go by the name of "idiots" or "naturals." The law of development demands of men the highest possible degree of intellectual and moral training and perfection. He who falls short of this is one of the "unfit" whose extinction nature has decreed.

